# Activation of renal epithelial Na^+^ channels (ENaC) in infants with congenital heart disease

**DOI:** 10.3389/fped.2024.1338672

**Published:** 2024-02-06

**Authors:** Laura A. Ortmann, Shyam Nandi, Yu-long Li, Hong Zheng, Kaushik P. Patel

**Affiliations:** ^1^ Department of Pediatrics, University of Nebraska Medical Center, Omaha, NE, United States; ^2^ Department of Integrative and Cellular Physiology, University of Nebraska Medical Center, Omaha, NE, United States; ^3^ Department of Emergency Medicine, University of Nebraska Medical Center, Omaha, NE, United States; ^4^ Basic Biomedical Sciences, Sanford School of Medicine, University of South Dakota, Vermillion, SD, United States

**Keywords:** ENaC, sodium retention, proteases, congenital heart disease, pediatric

## Abstract

**Introduction:**

This study was designed to measure the concentration and activity of urinary proteases that activate renal epithelial sodium channel (ENaC) mediated Na^+^ transport in infants with congenital heart disease, a potential mechanism for fluid retention.

**Methods:**

Urine samples from infants undergoing cardiac surgery were collected at three time points: T1) pre-operatively, T2) 6–8 h after surgery, and T3) 24 h after diuretics. Urine was collected from five heathy infant controls. The urine was tested for four proteases and whole-cell patch-clamp testing was conducted in renal collecting duct M-1 cells to test whether patient urine increased Na^+^ currents consistent with ENaC activation.

**Results:**

Heavy chain of plasminogen, furin, and prostasin were significantly higher in cardiac patients prior to surgery compared to controls. There was no difference in most proteases before and after surgery. Urine from cardiac patients produced a significantly greater increase in Na^+^ inward currents compared to healthy controls.

**Conclusion:**

Urine from infants with congenital heart disease is richer in proteases and has the potential to increase activation of ENaC in the nephron to enhance Na^+^ reabsorption, which may lead to fluid retention in this population.

## Impact

• Proteases known to activate ENaC were elevated in infants with congenital heart disease compared to healthy infants and their urine produced a significantly greater increase in Na^+^ inward currents in cortical collecting duct cells.
• There was no further increase in urinary proteases after cardiac surgery and protease levels were not associated with post-operative fluid overload.
• ENaC activate could be a contributor to pre-operative fluid retention in children with unrepaired congenital heart disease.

## Introduction

The incidence of fluid overload after surgery for congenital heart disease has been reported to be between 10% and 44% (1–3). Fluid overload is associated with longer ventilator times and longer hospital length of stays, and younger children are at higher risk (3–7). Despite its high incidence and impact on outcomes, the mechanisms underlying overload have not been fully investigated or described.

One potential mechanism for fluid retention in patients with congenital heart disease is the activation of renal epithelial Na^+^ channels (ENaC) in the convoluted tubules and collecting ducts of the distal nephron (8). ENaC activation increases permeability of the collecting duct to Na^+^, leading to sodium reabsorption which is followed by water (9–12). Cleavage of ENaC subunits by proteases in the tubular fluid is recognized as an important mechanism for ENaC activation (13). Increased levels of plasminogen, heavy chain of plasminogen, furin, and prostasin have been found in the urine of rats with heart failure and adults with congestive heart failure. This increase in proteases is possibly due to podocyte injury as demonstrated in rats with heart failure (14). Proteases have been shown to cleave extracellular domain of the ENaC subunits to activate the ENaC channel to enhance their function (15)*.* Furthermore, protease rich urine from rats and patients with heart failure has been shown to induce increased Na^+^ inward currents in renal cortical collecting duct (CCD) cells, suggesting proteolytic activation of ENaC may be a cause for Na^+^ and water retention in heart failure (14).

Interestingly, a high incidence of proteinuria is well described in children with heart disease (16, 17). However, the presence of specific urinary proteases that activate ENaC has not been reported or identified. Thus, this study has two objectives, first to measure the concentration of urinary proteases [plasminogen, heavy chain (plasminogen), furin, and prostasin] in infants with heart disease compared to healthy infants. Second, we aimed to assess whether urine from cardiac patients increased ENaC activation mediated Na^+^ currents in isolated renal CCD cells.

## Methods

### Patients with congenital heart disease

Infants less than 6 months of age who were undergoing cardiopulmonary bypass for repair or palliation of congenital heart disease were included, and were identified by examination of the surgery schedule and approached during their pre-operative evaluation. Exclusion criteria included patients with a Society of Thoracic Surgeons-European Association for Cardiothoracic Surgery mortality (STAT) score of 1 or 2 (18), a corrected gestational age <38 on the day of surgery, pre- or inter-operative extracorporeal membranous oxygenation use, acute kidney injury grade II (19) or higher in the 30 days before surgery, or a history of renal pathology. This study was approved by the institutional review board at the University of Nebraska Medical Center and informed consent was obtained from the legal guardian of all patients and control subjects.

**Figure 1:**
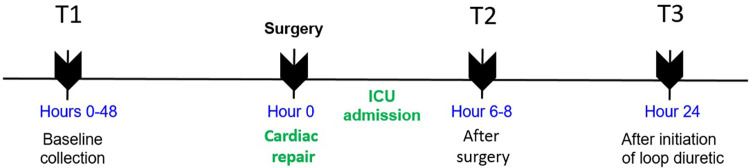
Schematic of urine sampling over time in this study.

Urine samples were obtained by catheter or bag at the following time points: T1, within 48 h prior to incision in the operating room; T2, 6–8 h after arrival in the cardiac intensive care unit (ICU) after surgery; and T3, 24 h after the initiation of loop diuretics. Timing of loop diuretic initiation was at the discretion of the clinical team. A schematic depicting the time of sampling is shown in Figure 1.

Baseline patient characteristics collected from the patient chart included age, weight, cardiac diagnosis, intravenous prostaglandin use, pre-operative loop diuretic use, cardiopulmonary bypass time, and cross clamp time. Presence of acute kidney injury (AKI) was assessed pre-operatively and at each time point according to the Kidney Disease Improving Global Outcomes creatinine classification: Stage 1, increase in creatinine of ≥50% or absolute increase in creatinine of 0.3 mg/dl; Stage 2, increase in creatinine of ≥100%; Stage 3, increase in creatinine of ≥200% and/or renal replacement therapy (19). Daily weights after surgery and total fluid intake and output were recorded for the first 48 h after surgery. Fluid overload was defined as an increase in weight by ≥10% at 24 and 48 h after surgery compared to the pre-operative weight.

### Control patients

Infants less than 6 months of age with no history of hospitalization, renal, and cardiac disease were consented to serve as controls. 10 ml of urine was collected by urine bag and tested for the four proteases of interest. Control subjects were recruited by a poster in the ICU breakroom.

### Urinary protease measured by immunoblotting

The concentrated urine samples from the control (*n* = 5) and patient groups (*n* = 18) were processed for Western immunoblot analysis. Each urine sample was filtered with 0.45 um filter paper and centrifuged at 4°C, 4,000 rpm for 30 min to collect the clear phase and exclude sediments. Filtered urine samples were concentrated using Amicon Ultra-15 Centrifugal Filter Unit (Millipore cat# UFC910024). Concentrated urinary protein samples were quantified using the Pierce BCA protein estimation assay kit. Total protein (25 mg from each sample) was mixed with an equal volume of 2× SDS sample buffer. The protein sample was then boiled for 5 min and loaded onto the 12% SDS-PAGE gel for electrophoresis at 70 volts for 4 h using 1× Tris/Glycine/SDS Running Buffer. The resolved proteins on the gel were electrophoretically transferred onto the polyvinylidene difluoride (PVDF) membrane (cat# IPFL07810, EMD Millipore) at 70 volts for 120 min in 1× Tris/Glycine Transfer Buffer. The transferred membrane was blocked for one hour with 5% non-fat dried milk (Biorad) in 1× Tris Buffered Saline solution at room temperature. In the next step, the transferred membrane was incubated with 1:1,000 diluted primary antibody [Anti-Plasminogen antibody cat# ab154560, Abcam; Prostasin/Prss8 Antibody, cat# H00005652-M11A, Novus Biologicals; Anti-Furin (B-6) cat# SC-133142, Santa-Cruz Biotechnology] overnight at 4°C. After washing, the membrane was incubated with secondary antibody as applicable (1:4,000 dilutions, Anti-mouse IgG, HRP-linked Antibody cat# 7076 or Anti-rabbit IgG, HRP-linked Antibody, Abcam cat# 7074 Cell Signaling Technologies) for two hours at room temperature. The chemiluminescent western blot band signals were visualized using a Thermo Scientific™ Pierce™ ECL Western Blotting Substrate (cat# PI32106) and detected with Bio-Rad chemiluminescent imager. Band intensity was quantified using ImageJ software (NIH).

### The effects of patient urine on ENaC activity in cultured renal CCD cells

M-1 mouse CCD cells (ATCC, Manassas, VA) were maintained in Dulbecco's Modified Eagle Medium: F12 (DMEM: F-12, cat# 30-2006, ATCC) supplemented with 5% Fetal Bovine Serum (cat# 30-2021, ATCC) and incubated at 37°C/5% CO_2_. For the experiment, cells were trypsinized and seeded to six-well plates and allowed to grow for 48 h in serum-free DMEM: F12 culture media for cell differentiation. Whole-cell patch-clamp recording was performed to measure the effects of protease-rich urine on ENaC activity.

This assay was conducted on single M-1 cells 24 h after seeding the cells onto cover slips and culturing them in serum-free medium using an Axopatch 200B patch-clamp ampliﬁer (Molecular Devices LLC, Sunnyvale, CA). The holding potential was −40 mV, and current-voltage (I–V) relationship was performed using voltage ramp from −100 mV to −20 mV over 200 ms. Current traces were sampled at 10 kHz and ﬁltered at 5 kHz. Inward currents were recorded before and after exposure to the urine collected and concentrated from controls and patients with or without ENaC inhibitor amiloride to determine Na^+^ currents.

### Statistical analysis

Demographic characteristics were described using median (interquartile range) or number and percent as appropriate. Differences in variables between male and female patients were assessed using Wilcoxon rank sum test for continuous variables or Fishers exact text for categorical variables. Pearson's correlation was used to test for correlations between continuous variable and protease levels. Differences between time points were tested with the student's paired *t*-test. Immunoblot intensity at T1 was compared between categorical variables using the student *t*-test. Pearson's correlation was used to assess correlation between protease levels and age. Analysis was performed using Statistical Analysis Software (version 9.4, SAS Incorporated, Cary, North Carolina). A *p*-value <0.05 was considered statistically significant.

## Results

### Baseline patient characteristics

Eighteen surgical patients and five controls were enrolled. One patient had a change in surgical plan after enrollment and did not undergo cardiopulmonary bypass so only T1 data was collected. Ten patients had urine collected at both T2 and T3 and are included in the post-operative analysis. Eight patients were missing either T2 or T3 urine due to missed or improper collection by the bedside staff. Median age of surgical patients was 0.2 (0.16–0.39) months and median age of controls was 2 (1.3–2.3) months (*p* = 0.06). Most patients (83%) were cyanotic with baseline oxygen saturations less than 85% before surgery, though only 17% continued to be cyanotic post-operatively. Other patient characteristics are listed in Table 1. Median time after surgery to first diuretic dose was 38 (24–44) hours and median time between first diuretic dose and T3 measurement was 24 (17–28) hours. No patients were diagnosed with AKI pre-operative or between surgery and T3.

**Table 1:** Baseline characteristics and clinical outcomes of patients undergoing surgery.

Characteristic	Study patients *n* = 18
Age, months	0.2 (0.16–0.39)
Weight, kg	3.5 (3.3–4.0)
Sex, male	11 (61)
Cardiac diagnosis^a^	
Atrioventricular canal	1 (6)
Left sided obstruction	3 (17)
Right sided obstruction	5 (28)
Total anomalous pulmonary vascular return	3 (17)
Transposition of the great arteries	3 (17)
Truncus arteriosus	3 (17)
Pre-operative cyanosis	15 (83)
Pre-operative prostaglandin infusion	8 (44)
Pre-operative loop diuretic use	5 (28)
Post-operative cyanosis	3 (17)
Cardiopulmonary bypass time, minutes	130 (113–170)
Cross clamp time, minutes	88 (57–102)
Hospital survival	16 (89)
Intensive care unit length of stay^b^, days	11 (6–21)
Hospital length of stay^b^, days	16 (11–34)

Data are presented as median (IQR) or number (percent) as appropriate.

^a^ Percentage does not equal 100 due to rounding.

^b^ Survivors.

### Urinary proteases were elevated in patients with congenital heart disease

**Figure 2:**
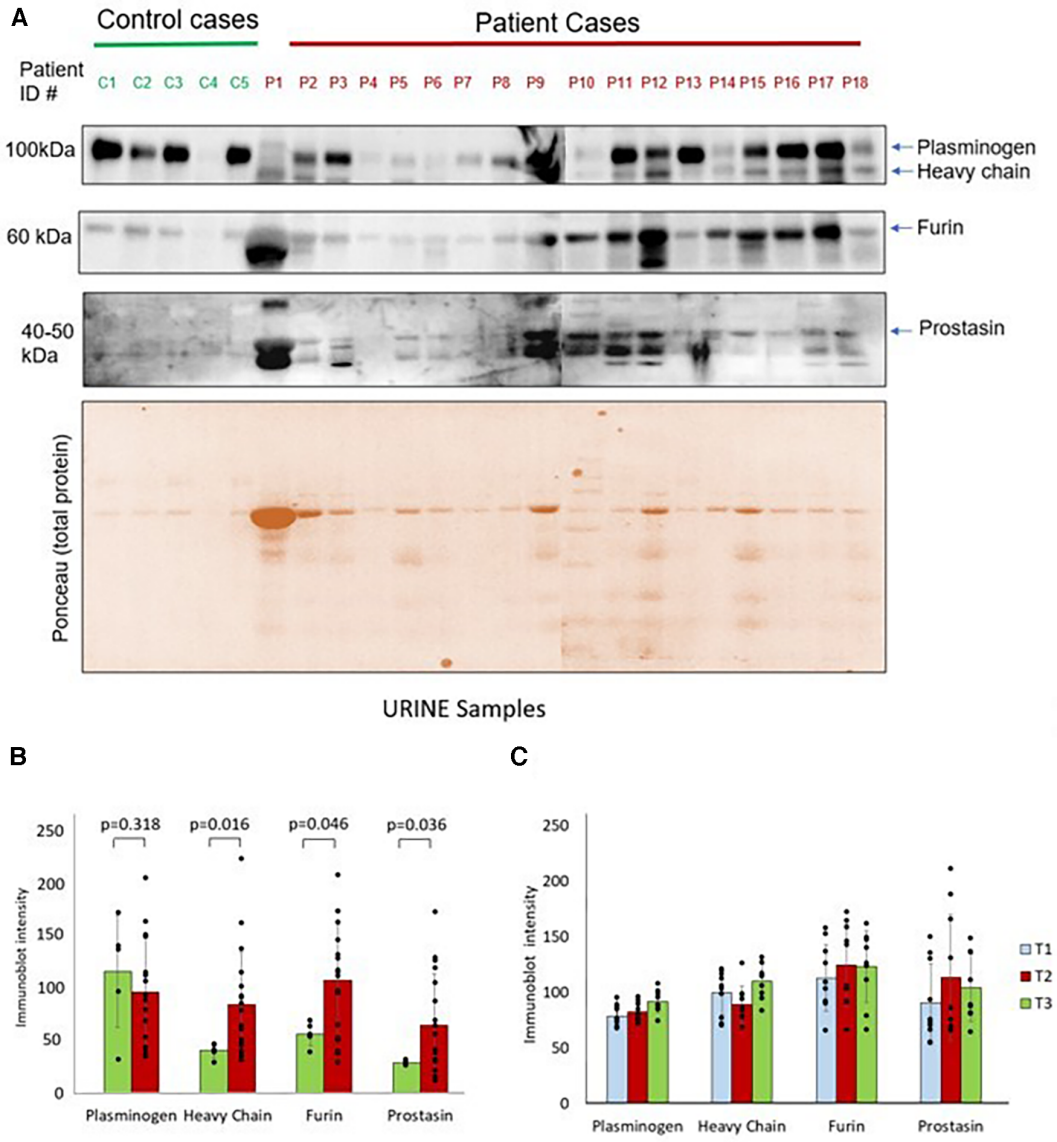
(**A**) Western blots show plasminogen, heavy chain, furin and prostasin protein expression in the urine from controls and surgical patients. Lower panel shows the ponceau staining image of the gel. (**B**) Protease immunoblot intensity in controls and surgical patients. Densitometry analyses of protease levels represented in arbitrary units. (**C**) Protease immunoblot intensity pre-operatively compared to post-operative time points. Difference between time points was significant between T2 and T3 for plasminogen (*p* = 0.02) and heavy chain (*p* = 0.03). All other comparisons: T1 vs. T2 and T2 vs. T3 had a *p-*value >0.05.

Western blots showing protease expression in the urine in control and pre-operative surgical patients are shown in Figure 2A. There were significantly elevated levels of heavy chain, furin, and prostasin in patients with congenital heart disease compared to healthy controls at T1 (Figure 2B). However, there was no significant difference in plasminogen between the groups at T1. There were no significant differences in immunoblot intensity in any of the four proteases between T1 and T2 (Figure 2C). There was a significant increase in plasminogen and heavy chain between T2 and T3. There were no significant differences in furin or prostasin between T2 and T3.

### Correlation between fluid overload and urinary proteases in patients with congenital heart disease

**Figure 3:**
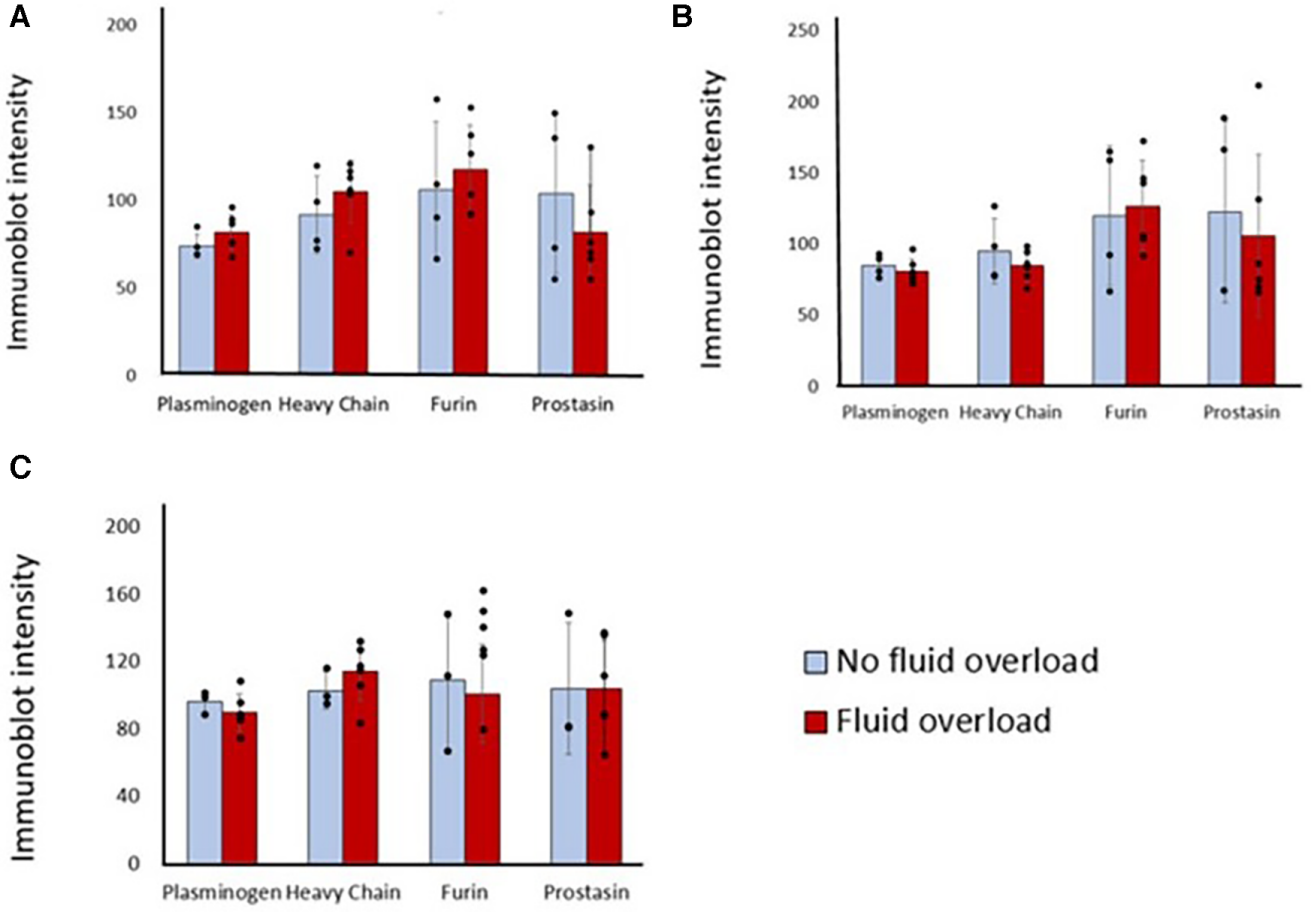
Differences in immunoblot intensity at each time point between those that were fluid overloaded 24 h after surgery vs. those that were not. Densitometry analyses of protease levels represented in arbitrary units. (**A**) T1, (**B**) T2, (**C**) T3. *P*-value is >0.05 between those that were fluid overloaded and those that were not at all time points.

Sixty percent of patients were fluid overloaded at 24 h post-operation and 80% were fluid overloaded at 48 h. There were no significant association between pre-operative or post-operative protease levels and the diagnosis of fluid overload at 24 h after surgery (Figure 3). Association testing was not done at 48 h due to the low number of patients without fluid overload at that time.

### Other associations of urinary proteases in patients with congenital heart disease

**Figure 4:**
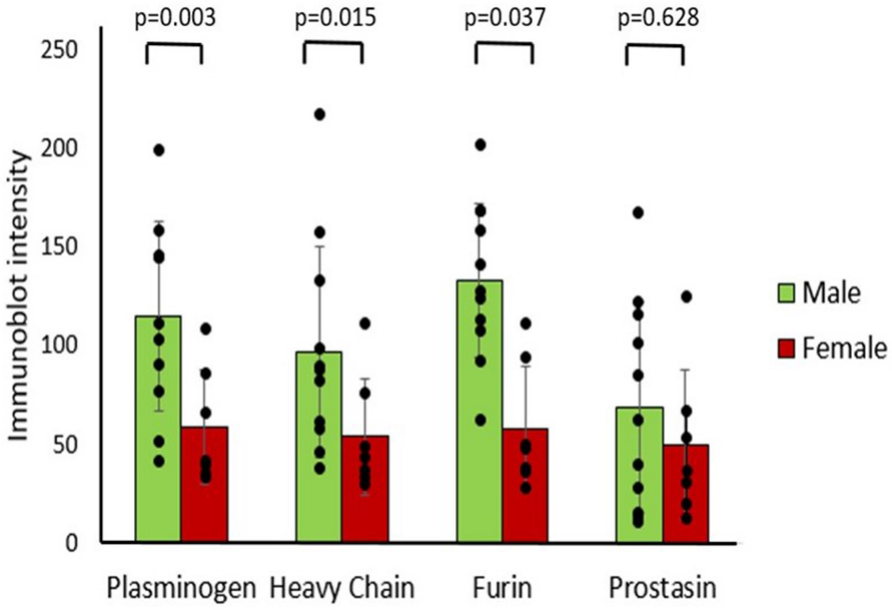
Differences in protease immunoblot intensity in males and females. Densitometry analysis of protease levels represented in arbitrary units.

There was no significant correlation between age and T1 immunoblot intensity (Pearson's correlations: plasminogen *r* = −0.331, heavy chain *r* = −0.362, furin *r* = −0.331, prostasin *r* = −0.028). Males had significantly higher immunoblot intensity at T1 in plasminogen, heavy chain, and furin but not in prostasin compared to females (Figure 4), though there were no demographic differences between sexes (Table 2). There was no correlation between T1 protease levels and the use of prostaglandin infusion or pre-operative diuretics (Supplementary Figure S1).

**Table 2:** Demographics between males and females.

	Male *n* = 11	Female *n* = 7	*p*-value
Age, months	0.2 (0.16–0.33)	0.3 (0.13–2.0)	0.682
Pre-operative cyanosis	10 (91)	5 (71)	0.682
Pre-operative prostaglandin infusion	5 (45)	3 (43)	0.538
Post-operative cyanosis	2 (18)	1 (14)	0.999
Collection method, catheter	4 (36)	4 (57)	0.387

Data are presented as median (IQR) or number (percent) as appropriate.

### Protease-rich urine activates ENaC in cultured renal CCD M-1 cells

**Figure 5:**
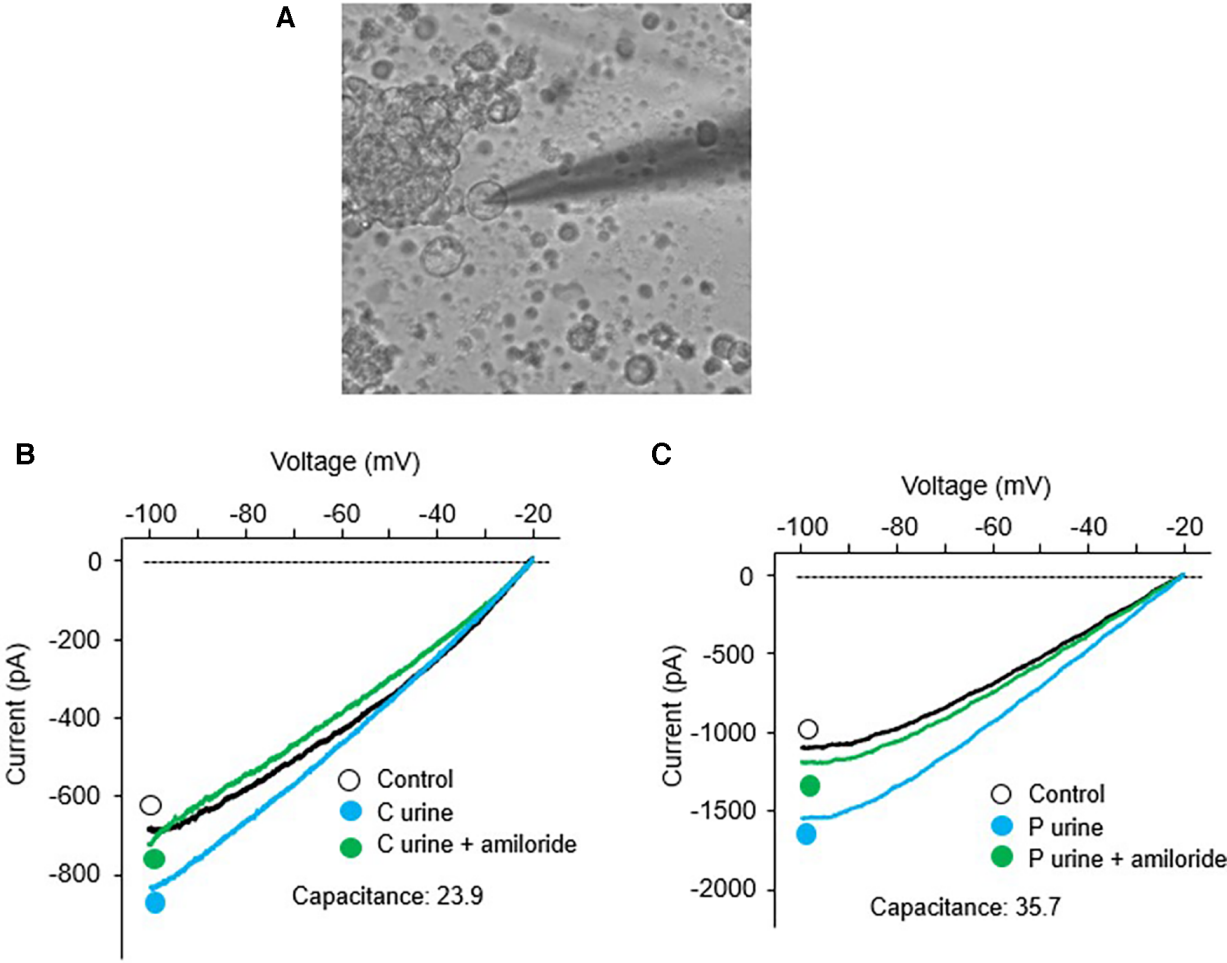
Whole-cell patch-clamp was conducted in M-1 cells; (**A**) A dipiction of whole cell patch recording setup. Recording of inward currents (voltage/current plots) before and after exposure to urine collected from (**B**) control patients with and without amiloride, and (**C**) surgical patients with and without amiloride.

**Figure 6:**
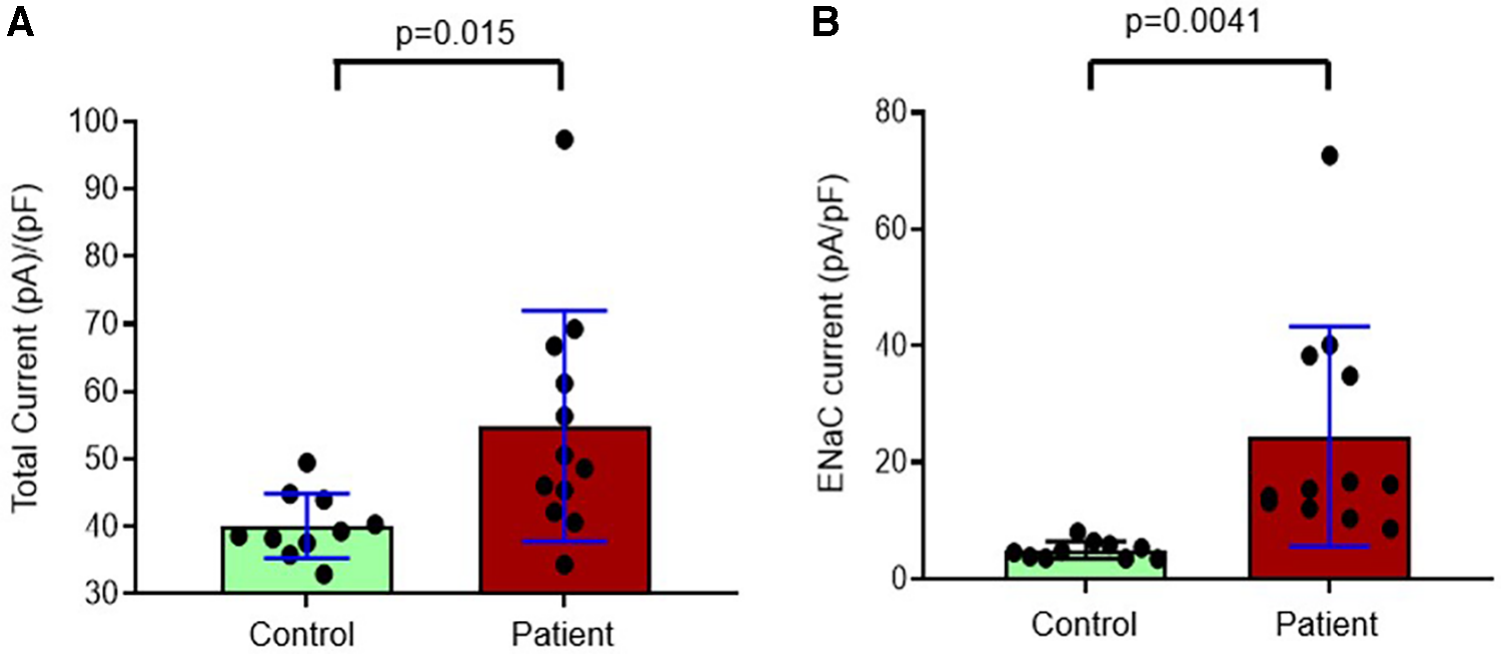
Composite data from whole-cell patch-clamp conducted in M-1 cells to record (**A**) total inward currents corrected for capacitance after exposure to urine collected from controls and patients with congenital heart disease and (**B**) eNaC currents corrected for capacitance in controls and patients with congenital heart disease.

Since we found significantly increased urinary proteases in patients with congenital heart disease, this study was designed to investigate the functional effects of protease-rich urine (from patients with congenital heart disease) on ENaC activity in cultured renal CCD M-1 cells. Whole-cell patch-clamp was conducted on M-1 mouse CCD cells to record inward currents before and after exposure to both control and patient urine (Figure 5A). Inward currents were decreased by the addition of the ENaC inhibitor amiloride, indicating the contribution due to Na^+^ current (Figures 5B,C). We have previously shown that electrophysiological alterations when M1 cells are incubated with urine from congestive heart failure rats in the presence of aprotinin nullified the ENaC current (14) indicating specificity of this measurement. The results show that protease-rich urine from patients with congenital heart disease increases the Na^+^ inward currents in M-1 cells compared to the control patients' urine, respectively (Figure 6).

## Discussion

Fluid retention before and after surgery can have a significant impact on symptoms and outcomes in congenital heart disease, but molecular mechanisms for this retention have not been well described in children. In this study, several urinary proteases that are associated with ENaC activation were higher in infants with congenital heart disease prior to undergoing surgery than in healthy controls. The protease rich urine from cardiac patients increased inward Na^+^ current in M-1 cells, signifying ENaC activation. Although surgery worsened fluid overload in patients with congenital heart disease there was no subsequent further increase in proteases in the urine of these patients. These data suggest increased Na^+^ reabsorption from urinary proteases may contribute to fluid overload in patients with unrepaired heart disease prior to surgery.

Children with congenital heart disease are at risk for congestive heart failure and volume overload due to left to right shunts, valve insufficiency, and diminished ventricular function. Up to half of children will require diuretics for decongestion prior to surgical repair (20). However, even children without overt symptoms of congestion have been found to have elevated total body water (21). As adults with congestive heart failure have been found to have increased urinary proteases as a potential mechanism for fluid overload (14), we hypothesized that similar mechanism may be operating in the case of children with congenital heart disease. There has been little research into the incidence and impact of fluid retention before surgery, with almost all investigation focusing on acute kidney injury and fluid overload after repair. Our study demonstrates that all four proteases, (plasminogen, heavy chain of plasminogen, furin, and prostasin) were lower in children receiving loop diuretics prior to surgery; however, there were only five children in the diuretic group and thus these differences did not reach statistically significance. It is possible that adequate decongestion in these patients led to improved end organ function and less renal injury that is thought to precipitate leaking of proteases in the urine.

Of note, pre-operative physiology varies widely in congenital heart disease and there may be differences in pre-operative fluid overload and proteases between lesions that could not be determined in this study due to the low number of each lesion type. We were also limited by low number of patients to assess whether pre or post-operative cyanosis was associated with protease levels. Less than six months of age was chosen for the inclusion criteria as younger infants are at higher risk for fluid overload, though most surgical patients in this study were less than 2 months of age. Urinary proteases have not been reported in infants, thus it is unknown how kidney maturation over the first several weeks and months of life may have influenced these results. A larger control group with younger infants could shed light on this question.

Interestingly, urinary proteases did not increase after surgery, despite a high prevalence of fluid overload. It is possible that cardiopulmonary bypass triggers a unique inflammatory response that results in decreased myocardial performance, increased systemic resistance, and leak of fluid from the vasculature into the tissues (22). Acute kidney injury, hemodynamic instability requiring fluid resuscitation, and large volumes of medications can all impact the incidence and degree of post-operative fluid overload. Given this complexity, renal ENaC activation *per se* may not have substantial effects on fluid retention in the first few days after surgery.

What remains unknown is whether ENaC activation becomes a significant factor in continued fluid overload after the bypass associated inflammatory reaction and/or acute kidney injury subside. The first post-operative sample (T2) was collected between 6 and 8 h after ICU admission which would correlate with the peak of post-operative inflammation. The second post-operative sample (T3) was collected at an average of 3 days after surgery. As median length of hospital stay was 21 days, T3 may have been too early to gauge the impact of continued increased ENaC activation on clinical status. Residual cardiac lesions are common after surgery and many children undergo palliative procedures rather than complete repairs. In fact, most children are discharged on diuretics (23), suggesting concern for continued fluid retention and a potential population and time frame for future study.

One noteworthy and unexpected finding in our investigation was that males had significantly higher urinary proteases than females prior to surgery. To our knowledge, sex differences have not previously been described for urinary protease concentrations. Differences in various outcomes in male infants are well known, with greater propensities for premature delivery and mortality from neonatal conditions (24, 25). Previous studies have also reported an increased risk for fluid overload in male infants after heart surgery (1, 26) but this may be due to the greater complexity of cardiac disease in males. Our observations of higher protease levels in male urine are consistent with a possible contribution of enhanced ENaC activity to the fluid overload observed in males. Further study is required to confirm these findings.

Urinary proteases' role in ENaC activation have been demonstrated in experimental models of heart failure (increased expression and activation) (14) and in nephrotic syndrome both by reduced sodium retention and inhibition of the expression of ENaC cleavage products during treatment with the protease inhibitor aprotinin (14, 27, 28). The impact of individual proteases in ENaC activation *in vivo* is still remains to be clarified. Interestingly sodium retention was not decreased in mouse model lacking urinary prostasin or plasmin (29–31), indicating further work needs to be done to understand the relationship between proteases and sodium retention in general as well as specifically in patients with proteinuria.

The M-1 cell line which is derived from the CCD of a transgenic mouse displays low-conductance and highly Na^+^ selective channel activity of the α, β, and γ-ENaC subunits. These cells retain several antigenic and differentiated transport properties of the CCD. These cells have been extensively used to investigate amiloride-sensitive Na^+^ transport through ENaC (32–34). In our patch clamp study in M-1 cells, the amiloride-sensitive, protease-activated inward Na^+^ current is consistent with ENaC activation as the link to Na^+^ and fluid retention, but results are not definitive. It is recognized that other amiloride-sensitive Na^+^ conducting channels such as ENaC-like channels and a non-selective cation channels cannot be irrefutably excluded by these particular experiments. In addition to our demonstration of increased inward current in MI cell with patient urine, it would be useful to demonstrate proteolytic cleavage of ENaC using WB of M1 cells after incubation with urine (and also after coincubation with aprotinin) to compliment the inward current data.

## Conclusion

Children with unrepaired congenital heart defects had increased urinary proteases and their urine induced increased inward Na^+^ currents in renal cortical collecting duct M-1 cells compared to urine of healthy infants. This increased ENaC activity may partly contribute to fluid retention and overload prior to surgical repair. This study provides a novel insight into one potential mechanism of the fluid overload commonly observed in congenital heart disease.

## Data Availability

The raw data supporting the conclusions of this article will be made available by the authors, without undue reservation.

## References

[1] Lex DJ Tóth R Czobor NR Alexander SI Breuer T Sápi E Fluid overload is associated with higher mortality and morbidity in pediatric patients undergoing cardiac surgery. Pediatr Crit Care Med. (2016) 17(4):307–14. 10.1097/PCC.0000000000000659 26914622

[2] Hassinger AB Wald EL Goodman DM. Early postoperative fluid overload precedes acute kidney injury and is associated with higher morbidity in pediatric cardiac surgery patients. Pediatr Crit Care Med. (2014) 15(2):131–38. 10.1097/PCC.0000000000000043 24366508

[3] Brandewie KL Selewski DT Bailly DK Bhat PN Diddle JW Ghbeis M Early postoperative weight-based fluid overload is associated with worse outcomes after neonatal cardiac surgery. Pediatr Nephrol. (2023) 38(9):3129–37. 10.1007/s00467-023-05929-7 36973562

[4] Seguin J Albright B Vertullo L Lai P Dancea A Bernier P-L Extent, risk factors, and outcome of fluid overload after pediatric heart surgery*. Crit Care Med. (2014) 42(12):2591–9. 10.1097/CCM.0000000000000517 25072753

[5] Wilder NS Yu S Donohue JE Goldberg CS Blatt NB. Fluid overload is associated with late poor outcomes in neonates following cardiac surgery. Pediatr Crit Care Med. (2016) 17(5):420–7. 10.1097/PCC.0000000000000715 27028790 PMC4856556

[6] Hazle MA Gajarski RJ Yu S Donohue J Blatt NB. Fluid overload in infants following congenital heart surgery. Pediatr Crit Care Med. (2013) 14(1):44–9. 10.1097/PCC.0b013e3182712799 23249789 PMC3668443

[7] Bailly DK Alten JA Gist KM Mah KE Kwiatkowski DM Valentine KM Fluid accumulation after neonatal congenital cardiac operation: clinical implications and outcomes. Ann Thorac Surg. (2022) 114(6):2288–94. 10.1016/j.athoracsur.2021.12.078 35245511 PMC9433462

[8] Zheng H Liu X Rao US Patel KP. Increased renal ENaC subunits and sodium retention in rats with chronic heart failure. Am J Physiol Renal Physiol. (2011) 300(3):F641–9. 10.1152/ajprenal.00254.2010 21159737 PMC4068120

[9] Rossier BC Canessa CM Schild L Horisberger JD. Epithelial sodium channels. Curr Opin Nephrol Hypertens. (1994) 3(5):487–96. 10.1097/00041552-199409000-00003 7804746

[10] Zachar RM Skjødt K Marcussen N Walter S Toft A Nielsen MR The epithelial sodium channel γ-subunit is processed proteolytically in human kidney. J Am Soc Nephrol. (2015) 26(1):95–106. 10.1681/ASN.2013111173 25060057 PMC4279735

[11] Kleyman TR Carattino MD Hughey RP. ENac at the cutting edge: regulation of epithelial sodium channels by proteases. J Biol Chem. (2009) 284(31):20447–51. 10.1074/jbc.R800083200 19401469 PMC2742807

[12] Rossier BC Stutts MJ. Activation of the epithelial sodium channel (ENaC) by serine proteases. Annu Rev Physiol. (2009) 71:361–79. 10.1146/annurev.physiol.010908.163108 18928407

[13] Hughey RP Carattino MD Kleyman TR. Role of proteolysis in the activation of epithelial sodium channels. Curr Opin Nephrol Hypertens. (2007) 16(5):444–50. 10.1097/MNH.0b013e32821f6072 17693760

[14] Zheng H Liu X Sharma NM Li Y Pliquett RU Patel KP. Urinary proteolytic activation of renal epithelial Na^+^ channels in chronic heart failure. Hypertension. (2016) 67(1):197–205. 10.1161/HYPERTENSIONAHA.115.05838 26628676 PMC4679598

[15] Passero CJ Carattino MD Kashlan OB Myerburg MM Hughey RP Kleyman TR. Defining an inhibitory domain in the gamma subunit of the epithelial sodium channel. Am J Physiol Renal Physiol. (2010) 299(4):F854–61. 10.1152/ajprenal.00316.2010 20630937 PMC2957262

[16] Krull F Ehrich JH Wurster U Toel U Rothgänger S Luhmer I. Renal involvement in patients with congenital cyanotic heart disease. Acta Paediatr Scand. (1991) 80(12):1214–9. 10.1111/j.1651-2227.1991.tb11811.x 1785294

[17] Hongsawong N Khamdee P Silvilairat S Chartapisak W. Prevalence and associated factors of renal dysfunction and proteinuria in cyanotic congenital heart disease. Pediatr Nephrol. (2018) 33(3):493–501. 10.1007/s00467-017-3804-3 28971258

[18] Jacobs ML O’Brien SM Jacobs JP Mavroudis C Lacour-Gayet F Pasquali SK An empirically based tool for analyzing morbidity associated with operations for congenital heart disease. J Thorac Cardiovasc Surg. (2013) 145(4):1046–57.e1. 10.1016/j.jtcvs.2012.06.029 22835225 PMC3824389

[19] Kellum JA Lameire N, KDIGO AKI Guideline Work Group. Diagnosis, evaluation, and management of acute kidney injury: a KDIGO summary (part 1). Crit Care. (2013) 17(1):204. 10.1186/cc11454 23394211 PMC4057151

[20] Rathgeber SL Chakrabarti A Kapravelou E Hemphill N Voss C Mammen C Association of preoperative diuretic use with early acute kidney injury in infants with biventricular hearts following cardiac surgery. J Am Heart Assoc. (2021) 10(20):e020519. 10.1161/JAHA.120.020519 34622667 PMC8751857

[21] Mitchell IM Davies PS Pollock JC Jamieson MP. Total body water in children with congenital heart disease, before and after cardiac surgery. J Thorac Cardiovasc Surg. (1995) 110(3):633–40. 10.1016/S0022-5223(95)70094-3 7564429

[22] Seghaye MC Grabitz RG Duchateau J Busse S Däbritz S Koch D Inflammatory reaction and capillary leak syndrome related to cardiopulmonary bypass in neonates undergoing cardiac operations. J Thorac Cardiovasc Surg. (1996) 112(3):687–97. 10.1016/s0022-5223(96)70053-3 8800157

[23] Trivedi M Diaz-Castrillon CE Morell E. Standardizing discharge furosemide duration following congenital heart surgery. World J Pediatr Congenit Heart Surg. (2022) 13(1):16–22. 10.1177/21501351211049308 34825593

[24] Khoury MJ Marks JS McCarthy BJ Zaro SM. Factors affecting the sex differential in neonatal mortality: the role of respiratory distress syndrome. Am J Obstet Gynecol. (1985) 151(6):777–82. 10.1016/0002-9378(85)90518-6 3976790

[25] Ingemarsson I Herbst A Thorngren-Jerneck K. Long term outcome after umbilical artery acidaemia at term birth: influence of gender and duration of fetal heart rate abnormalities. Br J Obstet Gynaecol. (1997) 104(10):1123–7. 10.1111/j.1471-0528.1997.tb10934.x 9332988

[26] Mah KE Hao S Sutherland SM Kwiatkowski DM Axelrod DM Almond CS Fluid overload independent of acute kidney injury predicts poor outcomes in neonates following congenital heart surgery. Pediatr Nephrol. (2018) 33(3):511–20. 10.1007/s00467-017-3818-x 29128923

[27] Bohnert BN Essigke D Janessa A Schneider JC Wörn M Kalo MZ Experimental nephrotic syndrome leads to proteolytic activation of the epithelial Na^+^ channel in the mouse kidney. Am J Physiol Renal Physiol. (2021) 321(4):F480–93. 10.1152/ajprenal.00199.2021 34423678

[28] Bohnert BN Menacher M Janessa A Wörn M Schork A Daiminger S Aprotinin prevents proteolytic epithelial sodium channel (ENaC) activation and volume retention in nephrotic syndrome. Kidney Int. (2018) 93(1):159–72. 10.1016/j.kint.2017.07.023 29042083

[29] Bohnert BN Daiminger S Wörn M Sure F Staudner T Ilyaskin AV Urokinase-type plasminogen activator (uPA) is not essential for epithelial sodium channel (ENaC)-mediated sodium retention in experimental nephrotic syndrome. Acta Physiol (Oxf). (2019) 227(4):e13286. 10.1111/apha.13286 31006168

[30] Xiao M Bohnert BN Aypek H Kretz O Grahammer F Aukschun U Plasminogen deficiency does not prevent sodium retention in a genetic mouse model of experimental nephrotic syndrome. Acta Physiol (Oxf). (2021) 231(1):e13512. 10.1111/apha.13512 32455507 PMC7688481

[31] Essigke D Ilyaskin AV Wörn M Bohnert BN Xiao M Daniel C Zymogen-locked mutant prostasin (Prss8) leads to incomplete proteolytic activation of the epithelial sodium channel (ENaC) and severely compromises triamterene tolerance in mice. Acta Physiol (Oxf). (2021) 232(1):e13640. 10.1111/apha.13640 33650216 PMC8159854

[32] Svenningsen P Bistrup C Friis UG Bertog M Haerteis S Krueger B Plasmin in nephrotic urine activates the epithelial sodium channel. J Am Soc Nephrol. (2009) 20(2):299–310. 10.1681/ASN.2008040364 19073825 PMC2637049

[33] Li K Guo D Zhu H Hering-Smith KS Hamm LL Ouyang J Interleukin-6 stimulates epithelial sodium channels in mouse cortical collecting duct cells. Am J Physiol Regul Integr Comp Physiol. (2010) 299(2):R590–5. 10.1152/ajpregu.00207.2009 20504903 PMC2928617

[34] Chalfant ML Peterson-Yantorno K O’Brien TG Civan MM. Regulation of epithelial Na^+^ channels from M-1 cortical collecting duct cells. Am J Physiol. (1996) 271(4 Pt 2):F861–70. 10.1152/ajprenal.1996.271.4.F861 8898016

