# Supplementary material for: Activation of renal epithelial Na+ channels (ENaC) in infants with congenital heart disease
Source: Front Pediatr. 2024 Feb 6;12:1338672. doi: 10.3389/fped.2024.1338672 (PMC10876900; doi:10.3389/fped.2024.1338672)

Supplemental Figure 1. Protease immunoblot intensity between patients with congenital heart disease that were on scheduled diuretics (n=5) prior to surgery and those who were not (n=13).

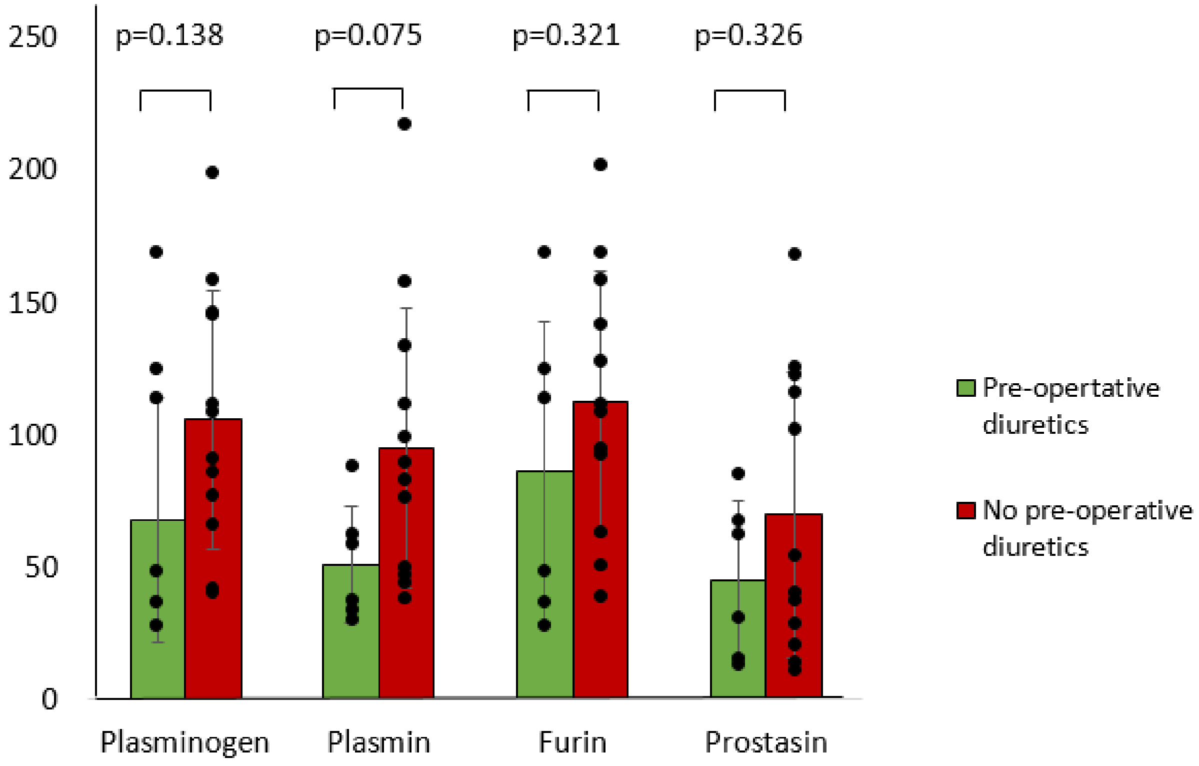

Supplement: Supplementary file 1 [file Image1.pdf]
